# Atopy-Dependent and Independent Immune Responses in the Heightened Severity of Atopics to Respiratory Viral Infections: Rat Model Studies

**DOI:** 10.3389/fimmu.2018.01805

**Published:** 2018-08-13

**Authors:** Jean-François Lauzon-Joset, Anya C. Jones, Kyle T. Mincham, Jenny A. Thomas, Louis A. Rosenthal, Anthony Bosco, Patrick G. Holt, Deborah H. Strickland

**Affiliations:** ^1^Telethon Kids Institute, University of Western Australia, Perth, WA, Australia; ^2^School of Medicine, University of Western Australia, Perth, WA, Australia; ^3^Department of Medicine, University of Wisconsin School of Medicine and Public Health, Madison, WI, United States

**Keywords:** rhinovirus, Th2-atopy, type I interferon, allergic asthma, microarray

## Abstract

Allergic (Th2^high^ immunophenotype) asthmatics have a heightened susceptibility to common respiratory viral infections such as human rhinovirus. Evidence suggests that the innate interferon response is deficient in asthmatic/atopic individuals, while other studies show no differences in antiviral response pathways. Unsensitized and OVA-sensitized/challenged Th2^high^ (BN rats) and Th2^low^ immunophenotype (PVG rats) animals were inoculated intranasally with attenuated mengovirus (vMC_0_). Sensitized animals were exposed/unexposed during the acute viral response phase. Cellular and transcriptomic profiling was performed on bronchoalveolar lavage cells. In unsensitized PVG rats, vMC_0_ elicits a prototypical antiviral response (neutrophilic airways inflammation, upregulation of Th1/type I interferon-related pathways). In contrast, response to infection in the Th2^high^ BN rats was associated with a radically altered intrinsic host response to respiratory viral infection, characterized by macrophage influx/Th2-associated pathways. In sensitized animals, response to virus infection alone was not altered compared to unsensitized animals. However, allergen exposure of sensitized animals during viral infection unleashes a notably exaggerated airways inflammatory response profile orders of magnitude higher in BN versus PVG rats despite similar viral loads. The co-exposure responses in the Th2^high^ BN incorporated type I interferon/Th1, alternative macrophage activation/Th2 and Th17 signatures. Similar factors may underlie the hyper-susceptibility to infection-associated airways inflammation characteristic of the human Th2^high^ immunophenotype.

## Introduction

Human rhinoviruses (HRV) are a broad family of respiratory viruses resulting in around 50% of respiratory tract infections (1) and are almost ubiquitous in children (2). While HRV infections are usually self-limiting, causing only mild “common cold” symptoms in the general population, HRV is a strong risk factor for asthma exacerbation in both adults and children (2, 3). Moreover, comparative studies tracking atopic versus non-atopic asthmatic school-age children across the autumn/winter virus season demonstrate that the intensification and spread of infections to the lower respiratory tract and ensuing loss of asthma control is more frequent among atopics (4). The mechanism(s) underlying the increased susceptibility of atopic asthmatics to severity and duration of symptoms associated with respiratory viral infections remain incompletely understood. Indirect evidence from human *in vitro* studies suggests that innate antiviral defense mechanisms (unrelated to atopic sensitization to allergen *per se*) may be altered/deficient in individuals expressing the Th2^high^ immunophenotype (5–8), while other studies report no differences between asthmatics and healthy controls (9–11). These discrepancies may be due to different study designs, or alternatively may be reflective of different endotypes of asthma (12–14).

Another important issue that requires further elucidation is the relationship between co-exposure to viral pathogen and aeroallergen in atopics. In co-exposed atopics, evidence suggests interactions between antiviral and Th2-associated effector mechanisms can result in enhanced Th2-mediated airways inflammation and concomitantly compromised viral clearance (15–17). These mechanisms are not mutually exclusive, but it is difficult to disentangle these co-factors in human clinical settings (18), which can only provide a snapshop of the disease.

Experimental animal models have thus been developed to unravel the detailed mechanisms with regards to how these risk factors (virus/atopy) intersect to potentially contribute to asthma exacerbations in the human situation. These studies have shown that HRV infection in Balb/c mice (Th2^high^ immunophenotype) induces an acute, short-lived prototypical antiviral inflammatory response (neutrophil influx/interferon), mainly driven by NFkB and PI3K-dependent pathways (19–22). Moreover, it has been clearly demonstrated in these models that during ongoing allergen-induced allergic airways inflammation, HRV infection potentiates the Th2 inflammatory component of the response, supporting a role for HRV in amplifying asthma exacerbation severity in humans (15–17). The mechanisms associated with enhanced Th2 response profiles include alarmins IL25 and IL33 (23, 24). There are some limitations associated with previous studies, including using only minor group HRV (19, 20, 25), the use of transgenic human ICAM-1 mice [as the majority of the HRV serotypes (HRV-A; 90%) require human ICAM-1 receptor for infection] (21, 26), the spectrum of susceptibility/severity observed in atopics and the relevance to potentially different phenotypes in asthma has not been adequately addressed (27, 28).

Recently, an alternative model of HRV infection has been developed using attenuated mengovirus (vMC_0_), a member of the picornavirus family, in both mice and rats (27, 28). vMC_0_ naturally infects rodents (29) and shares the same tropism for epithelial cells as HRV inducing a self-limiting inflammation of the airways (27, 30). We have used vMC_0_ to infect two strains of rats at opposing ends of the allergy spectrum (31–33) as a novel approach to understand the relationship between susceptibility/severity to viral infection and atopy (including allergen-(in)dependent interactions). BN rats are genetically at high-risk for atopic asthma and constitutively express the Th2^high^ immunophenotype, in contrast to the atopy-resistant Th2^low^ PVG strain. These strains exhibit high (BN) versus low (PVG) IgE production post-experimental sensitization and corresponding high versus low susceptibility to chronic Th2-associated airways inflammation post-aerosol challenge (32), recapitulating the clinical spectrum observed in sensitized humans.

## Materials and Methods

### Animals and vMC_0_ (Attenuated Mengovirus) Infection

BN and PVG rats were bred in-house at the Telethon Kids Institute (Perth, WA, Australia) under specified-pathogen-free conditions. All animal experiments were performed under guidelines from the National Health and Medical Research Council (NHMRC) of Australia. The institutional Animal Ethics Committee approved all procedures. 8- to 12-week-old male PVG and BN rats were utilized for all experiments. Attenuated mengovirus (vMC_0_) was prepared as previously described (28, 34) and rats were inoculated *via* intranasal (i.n.) administration with 100 µl of 10^7^ plaque-forming units (PFU) of vMC_0_ (27). The inflammatory response was assessed at day 1 post-infection (Dpi1) and Dpi3. The experimental asthma sensitization model involved sensitization of animals with 500 µl OVA/alum 14 days prior to vMC_0_ infection, followed by aerosol challenge (Ultraneb; DeVilbiss, Somerset, PA, USA) with 1% OVA (Sigma Aldrich, St. Louis, MO, USA) for 30 min at Dpi1 and assessment of the inflammatory response at Dpi 2 (24 h post-OVA challenge).

This study was carried out in accordance with the recommendations of NHMRC of Australia guidelines. The protocol was approved by the Telethon Kids Institute animal ethic committee.

### Bronchoalveolar Lavage (BAL) Processing and RNA Isolation

Bronchoalveolar lavage was performed with 8 ml GKN/5% FCS. Cell types were identified using Diff-Quick staining of cytocentrifuged samples. BAL cells were resuspended in RNAlater (Ambion, Life Technologies, Mulgrave, VIC, Australia) and frozen at −80°C. Total RNA was extracted from BAL cells with TRIzol (Ambion) followed by RNAeasy (Qiagen Gmbh, Hilden, Germany). The quality of the RNA was 8.8 ± 0.7 RIN (mean ± SD) as assessed on the Bioanalyzer (Agilent, Santa Clara, CA, USA).

### Viral Titre

Viral titre was measured in lung homogenates by plaque assay, as previously described (27). Briefly, monolayers of Hela cells were incubated with the samples for 30 min before adding an agar overlay (0.9%). 48 h later, cells were fixed with 4% formaldehyde and the number of PFU were counted per gram of tissue.

Viral copy number in BAL cells was confirmed by real-time quantitative PCR (RT-qPCR). The vMC_0_ primer sequence was, forward primer: 5′-GCC GAAAGC CAC GTG TGT AA and reverse primer: 5′-AGA TCC CAG CCA GTG GGG TA (35).

### Molecular Profiling

Total RNA extracted from BAL samples (*n* = 12) was labeled and hybridized to Affymetrix Rat Gene 2.1 ST 24-Array Plate. The raw microarray data are available from the Gene Expression Omnibus repository (GEO accession; GSE98152). The gene expression data were analyzed in R statistical programming language. The quality of the raw microarray data was assessed using ArrayQualityMetrics (36). The raw expression data were pre-processed with the robust multi-array average algorithm (37), which performs background correction, log2 transformation and quantile normalization. A custom chip description file (ragene21strnentrezgcdf, Version 19) was employed to annotate probe sets to genes based on updated genome information (38). The data were filtered with the proportion of variation accounted by the first principal component (PVAC) algorithm (39) to remove noisy probe sets from the analysis. Differentially expressed genes were identified employing moderated *t*-statistics LIMMA (40), with a false discovery rate control for multiple testing. Genes were deemed significant at adjusted *p*-value < 0.1. Pathways analysis was performed with Enrichr (41). Upstream regulator analysis (IPA) was performed with Ingenuity Systems software to identify putative molecular drivers of the observed gene expression patterns (42). IPA is based on prior knowledge of cause and effects between transcriptional regulators and target genes (e.g., protein–protein interactions).

### Real-Time Quantitative PCR

500 ng of total RNA from independent BAL samples 24 post-infection were utilized for reverse transcription into cDNA with the Quantitect Reverse Transcription Kit (Qiagen). The cDNA product was diluted 1:10 for quantitative real-time PCR analysis on the ABI 7500HT Real Time PCR System (Applied Biosystems). Differential expression of Th1 cytokines (*MX1, ISG15, IRF7*, and *CXCL10*), macrophage alternative activation (M2) genes (*MGL1, ARG1*, and *IL10*), and Th2 genes (*IL33* and *IL25*) identified in the microarray analysis were confirmed by RT-qPCR. The vMC_0_ primer was obtained from Sigma Aldrich and all other RT^2^ qPCR primer assays were obtained from Qiagen. The data were normalized to the housekeeping gene actin beta (*ACTB*) and relative gene expression levels were determined. Standard curves were generated by serial dilutions of known amounts of amplified cDNA.

### Statistical Analysis

Statistical analyses were performed using GraphPad Prism software (version 6.0g for Mac OSX, La Jolla, CA, USA). Non-parametric Kruskal–Wallis tests followed by Dunn’s multiple comparisons tests were used as indicated in the figure legends. Power of 80% was achieved with *n* = 3–6, for effect size ranging from 1.8 to 3. *P*-values < 0.05 were considered significant.

## Results

### vMC_0_ Infection in PVG and BN Rats: Lung Cell Infiltration Versus Viral Clearance

**Figure 1:**
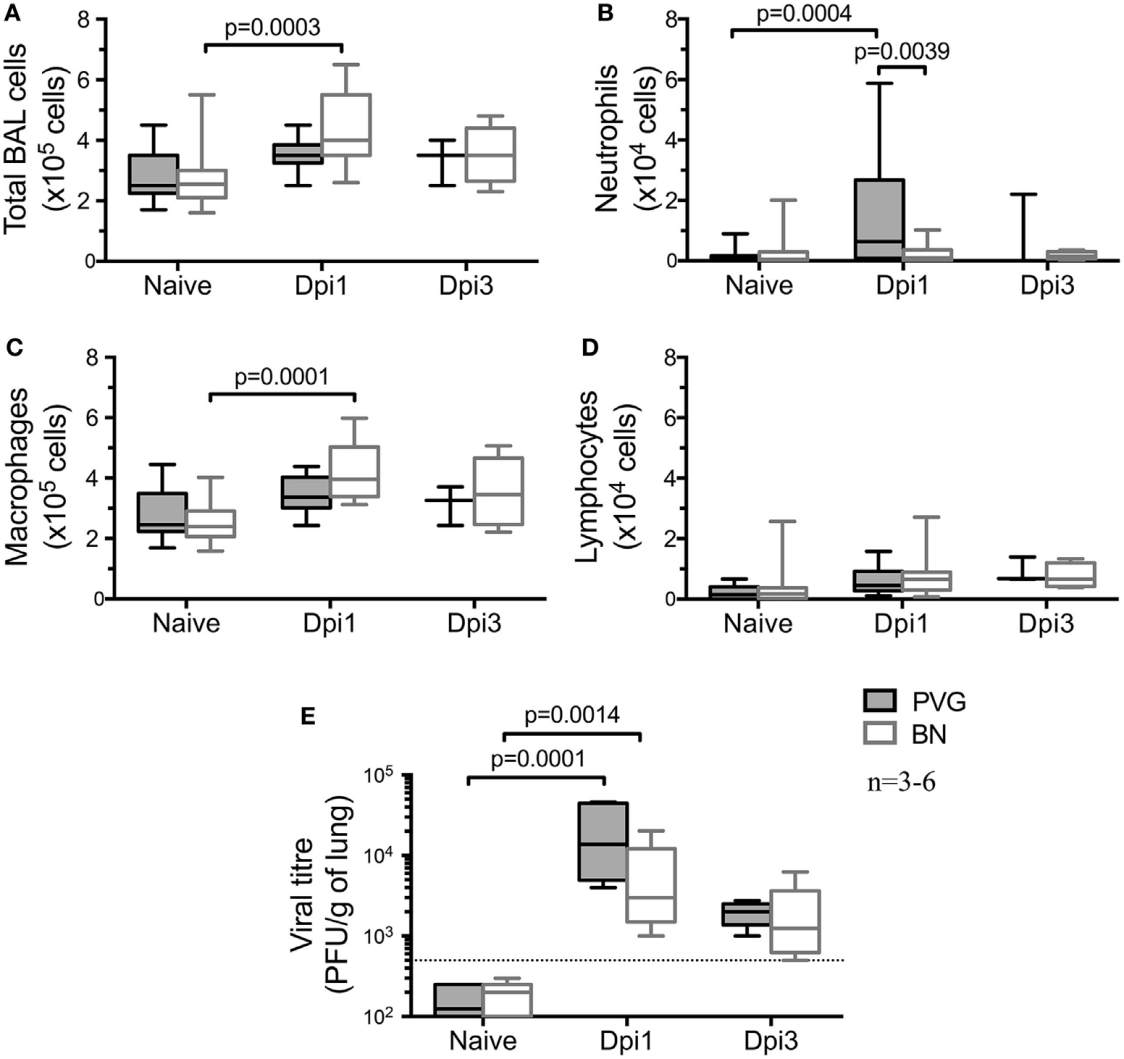
vMC_0_-induced airways inflammation in PVG and BN. Bronchoalveolar lavage (BAL) was obtained from naïve PVG (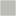) and BN (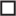) at one and 3 days post-vMC_0_ infection (dpi). **(A)** BAL total cell count, as well as **(B)** neutrophils, **(C)** macrophages, and **(D)** lymphocytes were enumerated using trypan blue and Diff-Quick staining. **(E)** Viral load was assessed in BAL of PVG and BN by plaque assay (*n* = 3–6). Statistical analysis was carried out by non-parametric Kruskal–Wallis test followed by Dunn’s multiple comparisons [overall *p*-values for panels **(A–E)** are respectively: 0.0002; 0.0095; 0.0001; 0.0856; and 0.0001].

We first investigated the default immune response to virus in the absence of sensitization in these Th2^high^/Th2^low^ strains. Assessment of the cellular composition in the airways by BAL at selected time points after vMC_0_ infection (Figures 1A–D) showed an acute cellular inflammatory response in both strains at day 1 post-vMC_0_ infection (Dpi1) that had largely resolved by Dpi3. Total BAL cellularity at Dpi1 was significantly increased in BN, whereas in PVG we only observed a trend (Figure 1A). However, there was a clear disparity in the profile of the cells recruited to the airways following viral infection. In PVG rats, vMC_0_ infection induced a significant early neutrophil influx in the BAL at Dpi1, which was resolved by Dpi3 (Figure 1B). In contrast, BN rats developed a significant transient influx of macrophages at Dpi1 (Figure 1C), with a notable absence of neutrophil recruitment, which is a hallmark feature of the normal host response to viral infection (Figure 1B). No significant lymphocyte recruitment (Figure 1D), nor eosinophil recruitment (data not shown) was observed in either strain. vMC_0_ viral load mirrored the early cellular response, peaking at Dpi1 in both strains and resolving by Dpi3 (Figure 1E). In both strains viral titres peaked on Dpi1, and while virus was detectable at that time point in all BN tested, titres were lower than PVG (Figure 1E). No virus was detected in naïve rats.

### Molecular Profiling of BAL Cells From PVG and BN Rats Following vMC_0_ Infection

**Figure 2:**
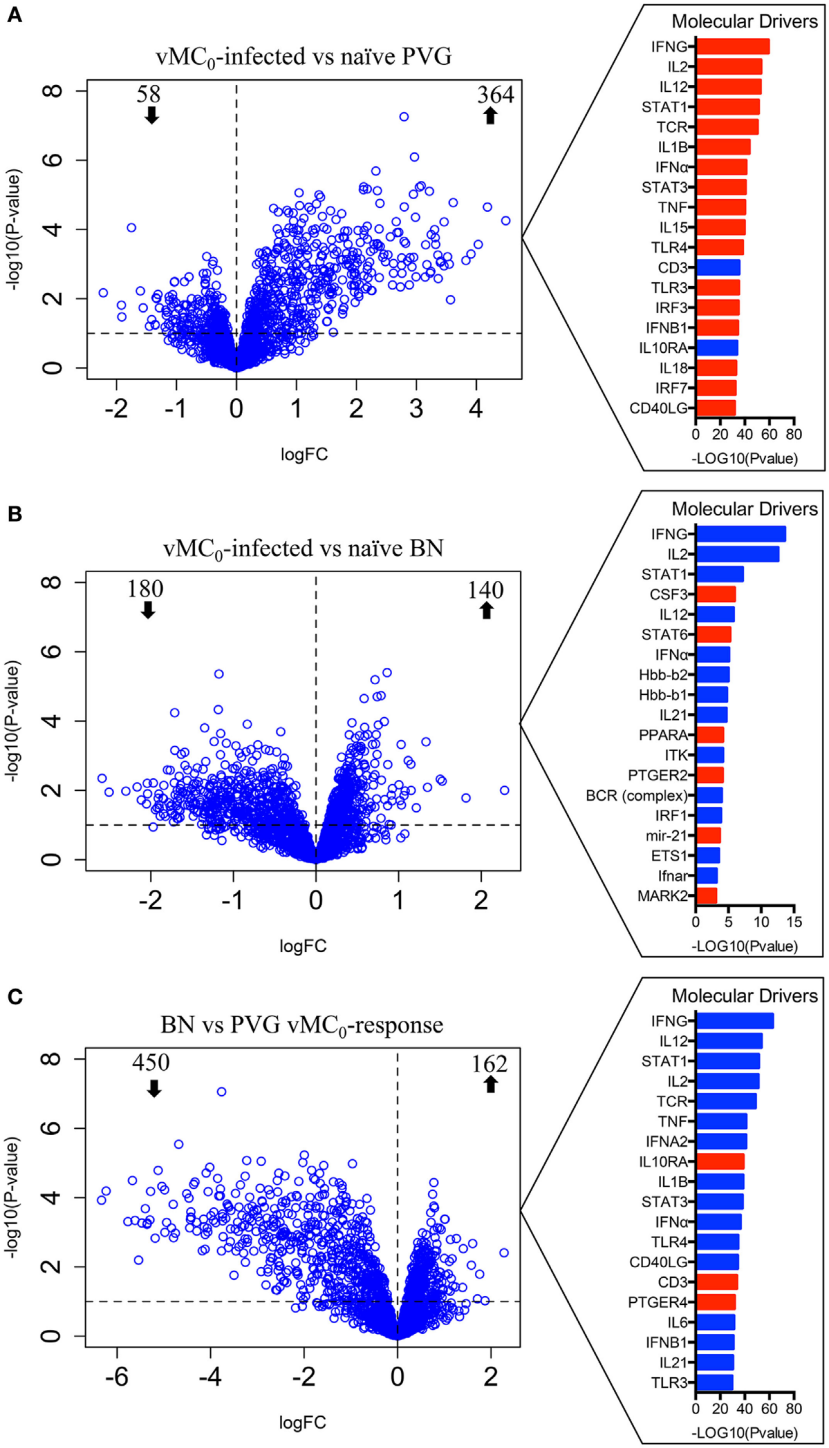
vMC_0_ infection induces an antiviral response in Th2^low^ PVG, but not in the Th2^high^ BN. Bronchoalveolar lavage (BAL) cells were collected in PVG and BN at day 1 post-infection. Left panel: differentially expressed genes of BAL cells were identified comparing **(A)** naïve versus vMC_0_-infected PVG rats, **(B)** naïve versus vMC_0_-infected BN rats, and **(C)** BN versus PVG responses to vMC_0_. The dashed horizontal lines indicate an adjusted *p*-value <0.1; *n* = 3. Right panel: top molecular drivers were identified with the Ingenuity Knowledge Base. Drivers in red denote activation and blue indicates inhibition. Absolute activation *Z*-scores ≥2.0 and *p*-values <0.01 were deemed significant.

To elucidate the molecular mechanisms underlying these divergent antiviral responses, gene expression profiles of airway inflammatory cells in BAL were measured at Dpi1. vMC_0_ infection of Th2^low^ PVG rats induced a strong perturbation of the gene expression program, altering the expression of 422 genes (364 upregulated and 58 downregulated) (Figure 2A; Table S1 in Supplementary Material). Employing upstream regulator analysis, we identified Th1 cytokines (*IFNG, IL2*, and *IL12*) and a type I interferon (*IFN*) response (*STAT1* and *IFNA*), as the top predicted molecular drivers of the PVG response to virus (Figure 2A). Pathways analysis showed that the viral response of PVG rats was associated with upregulation of T cell activation, cell migration, and antiviral responses (Table S2 in Supplementary Material, *p* = 10^−14^–10^−29^). In Th2^high^ BN rats, viral infection resulted in the modulation of 320 genes at Dpi1 (140 upregulated and 180 downregulated) (Figure 2B; Table S3 in Supplementary Material). Strikingly, subsequent driver analysis of the expression patterns in the BN response demonstrated downregulation of genes downstream of the Th1-trophic cytokines, which were identified in Figure 2A as the strongest drivers of the antiviral response in the PVG strain, with concomitant activation of Th2 pathways (*CSF3, STAT6*, and *mir-21*; Figure 2B). Pathways analysis demonstrated downregulation of cell adhesion and T cell activation (Table S4 in Supplementary Material, *p* = 10^−3^–10^−9^) in the BN. We then performed a direct comparison of the vMC_0_-response between the two strains, and identified 162 genes with higher expression and 450 genes with lower expression in the BN versus the PVG responses (Figure 2C; Table S5 in Supplementary Material). Upstream driver analysis of the respective responses revealed higher expression of alternative macrophage activation (M2)/Th2-associated genes (*IL10RA* and *PTGER4*) and lower expression of multiple Th1-associated genes in the BN (Figure 2C; Table S6 in Supplementary Material, *p* = 10^−23^–10^−48^).

**Figure 3:**
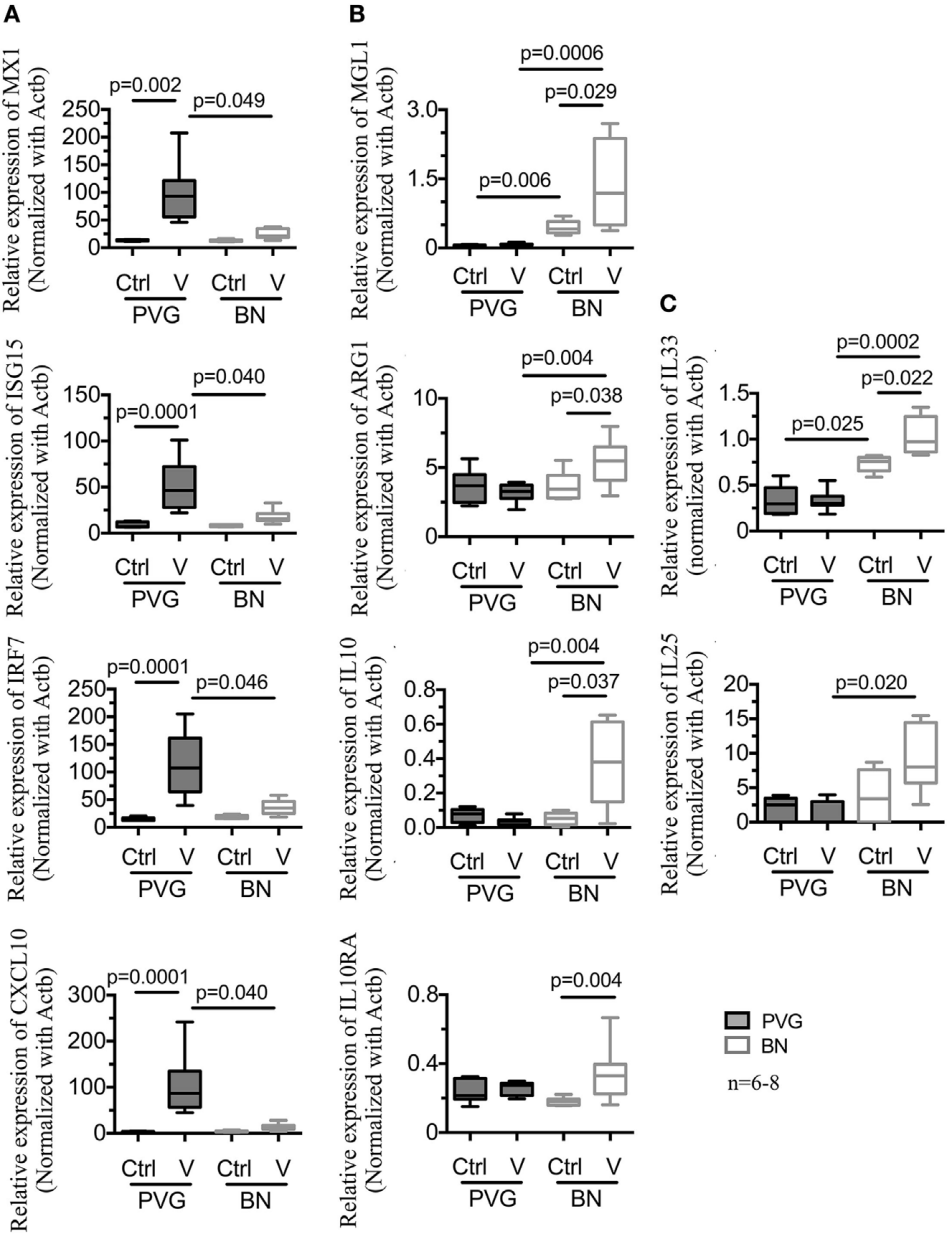
vMC_0_ infection in BN is characterized by an alternative activation of macrophages and Th2 response contrary to an archetypal type I interferon response. Total RNA was obtained from independent bronchoalveolar lavage samples of PVG (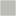) and BN (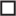) rats at day 1 post-infection. Real-time quantitative PCR was carried out on a subset of genes representative of the main pathways identified in Figure 2. **(A)** Viral infection induced the expression of type I interferon and antiviral response genes in PVG, whereas in BN, we observed an increased expression of **(B)** alternative activation of macrophages and immunoregulatory genes, and **(C)** Th2 response genes. *n* = 6–8; statistical analysis was carried out by non-parametric Kruskal–Wallis test followed by Dunn’s multiple comparisons (overall *p*-values, MX1: 0.0001; ISG15: 0.0001; IRF7: 0.0001; CXCL10: 0.0001; MGL1: 0.0001; ARG1: 0.0265; IL10: 0.028; IL10RA: 0.0328; IL33: 0.0002; and IL25: 0.0295).

To validate the above findings, a subset of genes representative of the main pathways was selected for RT-qPCR experiments using BAL samples from an independent set of vMC_0_-infected animals. Confirming our initial findings, viral infection of PVG rats increased the expression of Th1 and type I IFN target genes (*MX1, ISG15, IRF7*, and *CXCL10*) (Figure 3A). Our initial findings were also validated in vMC_0_-infected BN rats, where infection resulted in the induction of genes associated with macrophage M2 activation (*MGL1, ARG1, IL10*, and *IL10RA*) and Th2 genes (*IL33* and *IL25*) (Figures 3B,C). Of note, relative levels of *MGL1* and *IL33* at baseline were higher in BN compared to PVG, and this difference was further amplified following infection (Figures 3B,C). No differences in gene expression were observed in either strain for *NOS2, TNF, IL1B*, and *LY6C* following vMC_0_ infection (data not shown).

### Co-Exposure to vMC_0_ and Aeroallergen

**Figure 4:**
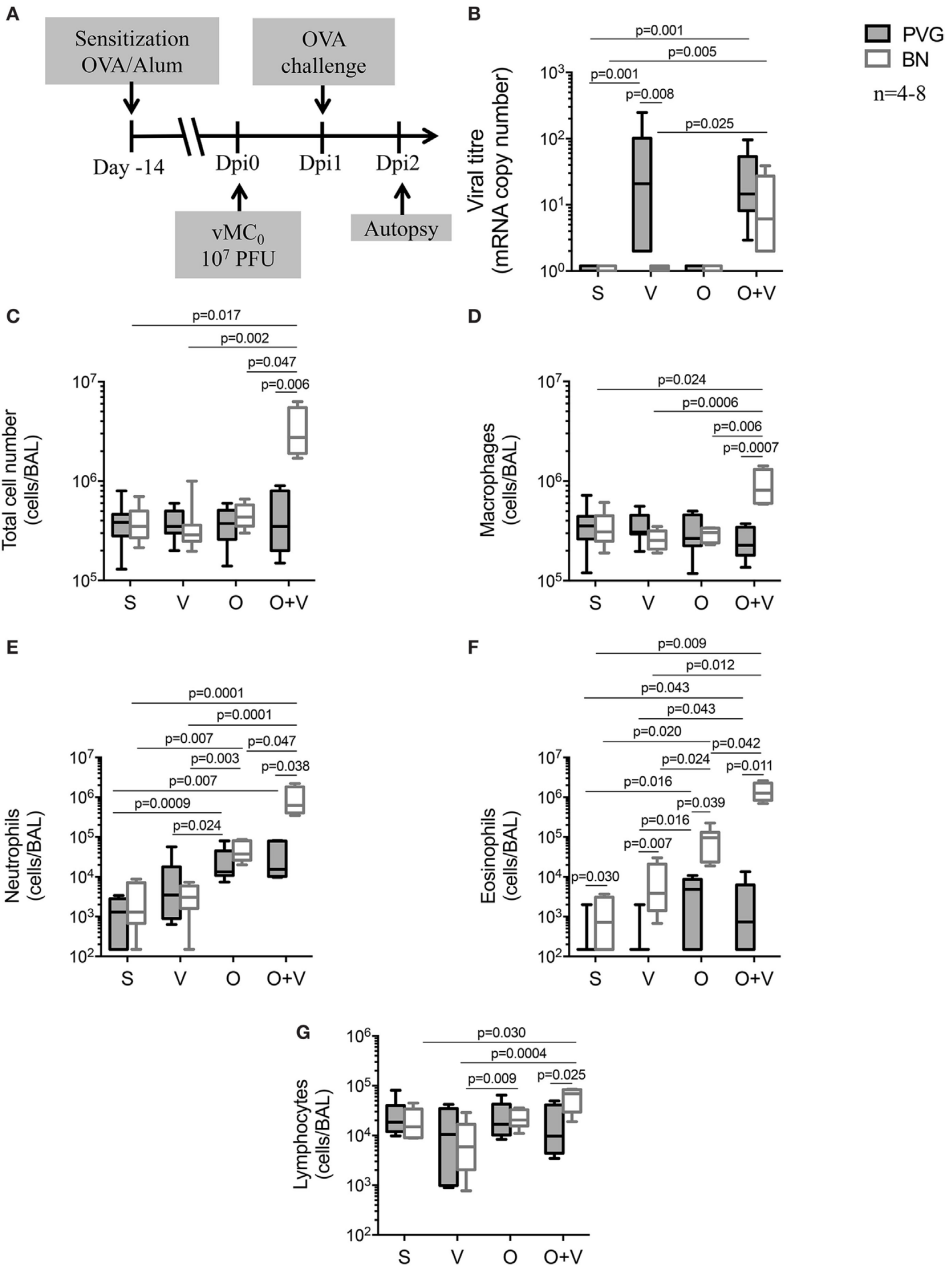
vMC_0_ infection prior to allergen exposure triggers severe airways disease in BN rats. **(A)** PVG (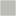) and BN (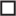) rats were sensitized (S) with OVA/alum 14 days prior to vMC_0_ infection (V), OVA allergen challenge (O) at day 1 post-infection (Dpi1). **(B)** Viral titres were measured by real-time quantitative PCR. Bronchoalveolar lavage were collected at Dpi2 and **(C)** total cell numbers were measured in BAL, as well as **(D)** macrophages, **(E)** neutrophils, **(F)** eosinophils, and **(G)** lymphocytes. *n* = 4–8; statistical analysis was carried out by non-parametric Kruskal–Wallis test followed by Dunn’s multiple comparisons [overall *p*-values for panels **(B–G)** are respectively: 0.0001; 0.030; 0.025; 0.0001; 0.0001; and 0.025].

In order to model the effects of concomitant virus and aeroallergen exposure in a context relevant to sensitized humans, PVG and BN rats were pre-sensitized to OVA allergen prior to infection with vMC_0_ (Dpi0) (27), and subsequently exposed to OVA aerosol 24 h later (Dpi1) (32). BAL samples were collected 24 h post-OVA challenge (Dpi2) (Figure 4A). As expected and previously reported (32), BN OVA-specific IgE titres were higher than PVG (data not shown). Viral infection alone or co-exposure to virus and allergen did not significantly alter OVA-specific IgE titres (data not shown). Viral titres (Figure 4B) in sensitized Th2^high^ BN rats dramatically increased with concomitant viral and allergen challenge compared to virus only, whereas in Th2^low^ PVG rats, viral plus allergen exposure did not alter lung viral titres compared to virus alone.

Bronchoalveolar lavage total cell numbers (Figure 4C) and inflammatory cell recruitment were also assessed (Figures 4D–G). Sensitization *per se* did not modulate the acute response to vMC_0_ (Figure S1 in Supplementary Material), nor did it impact the resolution of inflammation, as observed in the virus only exposure (Figure 4; corresponding to Dpi2) showing only minor changes in BAL cellular composition of both PVG and BN (Figure 4). Allergen exposure of sensitized PVG triggered the recruitment of neutrophils, with a small eosinophil component, whereas in BN the response was dominated by the recruitment of eosinophils, with a neutrophilic component (Figure 4), as observed previously (31–33). The presence of an underlying viral infection did not alter the PVG response to the allergen (Figure 4). In contrast, allergen exposure in virus-infected sensitized BN elicited a much stronger inflammatory response with significant increases of macrophages, neutrophils, and eosinophils, and to a lesser extent, lymphocytes (Figure 4). Strikingly, the neutrophilic and eosinophilic response to virus and allergen in BN represents a 20- and 16-fold increase, respectively, compared to allergen alone (Figure 4).

To further elucidate the temporal relationship between viral infection and allergen sensitization/exposure, allergen exposure was carried out on different days post-viral infection in sensitized rats (Dpi1, 2, and 3; Figure S2A in Supplementary Material). Severe airways inflammation was only observed when allergen exposure was shortly after the viral infection (Dpi1 and, to a lesser extent Dpi2; Figure S2B in Supplementary Material).

**Figure 5:**
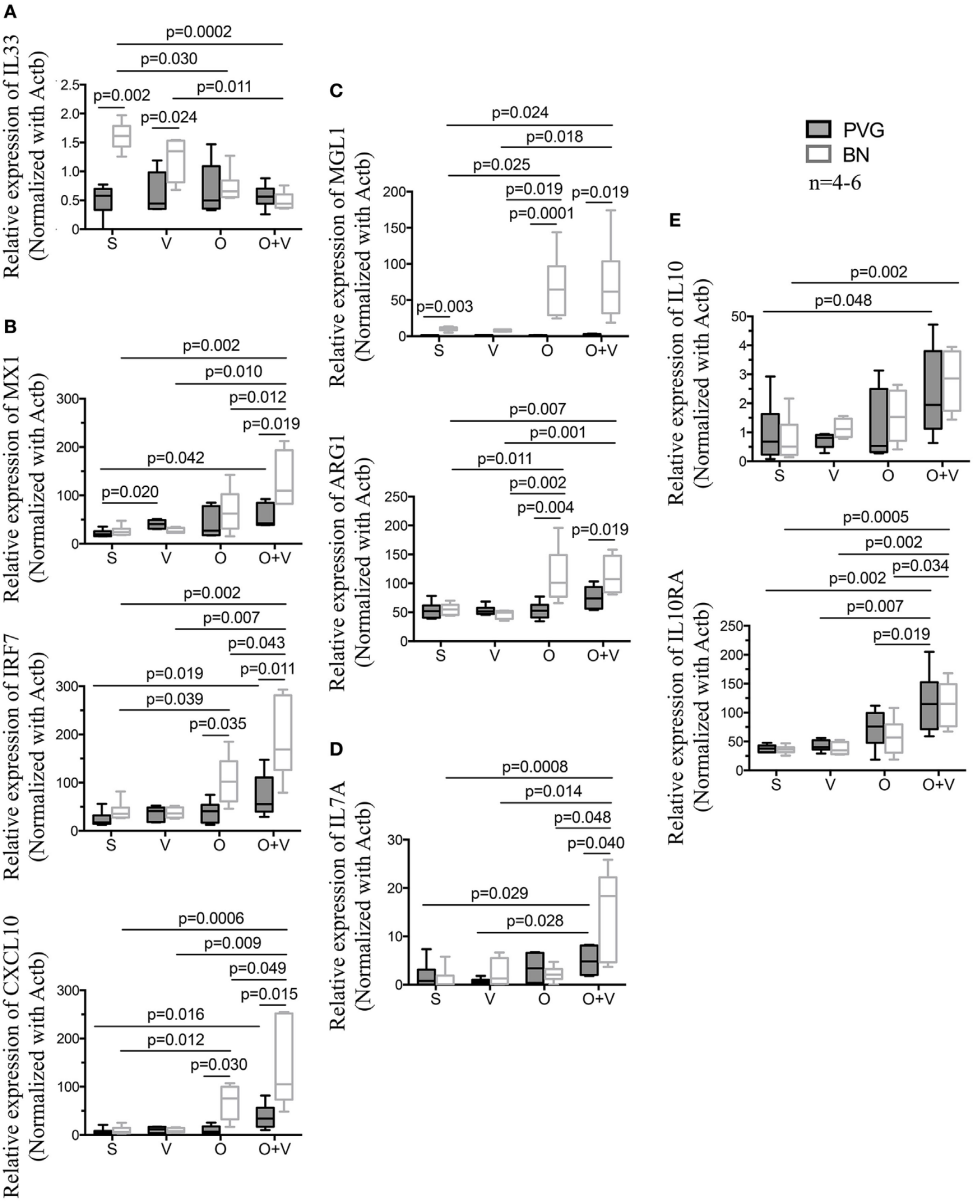
Co-exposure to virus and allergen induces a mixed type I IFN and Th2 gene signature in BN rats. PVG (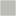) and BN (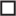) rats were sensitized (S) with OVA/alum 14 days prior to vMC_0_ infection (V), OVA allergen challenge (O) at day 1 post-infection (Dpi1). Bronchoalveolar lavage cells were collected at Dpi2 and total RNA was extracted. Real-time quantitative PCR of a subset of genes representative of the main pathways identified in Figure 2 was carried out. **(A)** IL33 gene expression; **(B)** Type I interferon and antiviral response genes; **(C)** alternative activation of macrophages; **(D)** IL17a; and **(E)** immunoregulatory genes. All animals were sensitized to OVA/alum. *n* = 4–6; statistical analysis was carried out by non-parametric Kruskal–Wallis test followed by Dunn’s multiple comparisons (overall *p*-values, IL33: 0.002; MX1: 0.009; IRF7: 0.0005; CXCL10: 0.0007; MGL1: 0.0001; ARG1: 0.0005; IL17A: 0.009; IL10: 0.016; and IL10RA: 0.0003).

Finally, we measured the gene expression of BAL cells by RT-qPCR from sensitized Th2^low^ PVG and Th2^high^ BN after exposure to virus, allergen, or both stimuli (Figure 5). Gene expression in sensitized animals exposed to virus only showed increased Th2/*IL33* expression in Th2^high^ BN versus Th2^low^ PVG (Figure 5A). In contrast, challenge of sensitized Th2^high^ BN to allergen alone resulted in increased Th1 (*IRF7* and *CXCL10*; Figure 5B) and M2 activation (*MGL1* and *ARG1*; Figure 5C). The most striking finding was in sensitized Th2^high^ BN co-exposed to virus and allergen resulting in further increase of Th1 gene expression (*IRF7, CXCL10*, and *MX1*; Figure 5B) and expression of Th17 (*IL17A*; Figure 5D). BN an PVG *IL10*/*IL10RA* expression was increased after virus and allergen exposure (Figure 5E). In addition, *IL33* and *MGL1* expression in sensitized BN was higher than in PVG (Figure 5), as observed in non-sensitized animals (Figure 3C).

## Discussion

Severe lower respiratory infections, particularly with HRV, can trigger asthma exacerbations requiring hospitalization, especially in atopics. It is unclear whether the default host response to virus (in the absence of sensitization) is aberrant in individuals genetically susceptible to atopy, or if sensitization/allergen exposure plays a direct role in the context of increased disease severity. Understanding the mechanisms that drive severe respiratory viral induced asthma exacerbations would significantly contribute toward the development of more effective therapeutic and/or preventive strategies. Here, we compared the responses of rats expressing Th2^low^ versus Th2^high^ immunophenotypes, to respiratory infection with vMC_0_ as a model for HRV infections, allergen alone, and concomitant vMC_0_/allergen, and assessed ensuing viral clearance and associated inflammatory responses. The initial phase of the antiviral response superficially appears to proceed more efficiently in BN as evidenced by lower titres of virus on day 1. Of note, however, this was associated with increased inflammatory cell accumulation in the airways that was dominated by macrophages, as opposed to the neutrophilic response in PVG, which is conventionally associated with rapid mobilization of antiviral immunity. Moreover, naive BN showed a Th2 bias following infection with vMC_0_, as shown by an influx of alternatively activated macrophages (*MGL1/ARG1*) with parallel upregulation of Th2-associated cytokines (*IL25/IL33*). Here, it is unclear whether lower viral titre in BN on day 1 may be attributable to more effective clearance or alternatively different disease kinetics in the two strains.

The differences between the airway inflammatory responses in these two strains become much more profound when concomitant aeroallergen challenge is superimposed on the initial viral infection, mimicking the common human situation of viral infection in human atopics sensitized to perennial indoor allergens. In particular, the BN manifest a rapid increase in viral titre, whereas this is unaffected in PVG. Moreover, BN display granulocytic responses dominated by eosinophils and to a lesser extent neutrophils, in numbers that are log-fold higher than responses to allergen or virus alone and in comparison to PVG. This rapid and intense inflammatory response in BN was associated with the expression of Th1/interferon, Th2, and Th17 inflammation. In contrast, the Th2^low^ PVG response to allergen challenge was unchanged with concomitant viral infection. Of note, the viral load in these animals was similar, suggesting that the total viral load is not driving the increased inflammatory response, but rather that in the context of the Th2^high^ immunophenotype, allergen, and virus-induced inflammatory pathways interact in airway tissues to amplify local inflammation. It is noteworthy that the current literature reports conflicting results concerning the capacity of human asthmatics to generate type I IFN responses to viral infection [deficiency versus normal expression compared to healthy controls (43–47)], but the contribution of underlying atopy to this heterogeneity has not yet been systematically investigated.

Our findings corroborate HRV1b and HRV16 mouse models post-allergen exposure (19, 25, 26), although in our model a single (acute) sensitization/allergen exposure was employed and notably, viral infection preceded allergen exposure (as opposed to multiple allergen exposures followed by a viral infection). In addition, allergen exposed BN had a high viral load in the lungs (Figure 4B; BN-V versus BN-OV), supporting the notion that viral load is increased in human asthmatics (6, 17, 48) and the combination of sensitization, viral infection, and allergen exposure confers the greatest risk for asthma exacerbation as reported for humans (49). Many pathways have been shown to interfere with viral clearance, including activation of the high-affinity receptor (FcεRI) for IgE and type 2 cytokines (15, 24, 48), but further studies are required to elucidate which pathway(s) are involved in the increased viral load after allergen exposure in BN. Overall, these findings reinforce the notion that viral infection with concomitant allergen exposure in sensitized individuals is a strong risk factor for asthma exacerbation and hospital admission (49).

It is additionally evident in BN that the combined effect of viral plus allergen exposure on the neutrophil and eosinophil components of these responses clearly exceeds the sum of those observed for each stimulus alone, suggesting synergistic interactions between the underlying inflammatory pathways. As shown by the RT-qPCR data in Figure 5, the latter pattern is mirrored by changes in expression of genes associated with type I IFN and IL17 pathways, which display marked upregulation unique to the dual exposure group. In contrast, parallel upregulation of genes exemplary of the (Th2-dependent) M2 activation pathway and accompanying downregulation of *IL33*, which is constitutively expressed at high-levels in the BN, appears to be a direct response to aeroallergen exposure alone. This is in contrast to other models (19, 26) where co-exposure triggered an increased expression of both Th2 and Th1/type I responses. This discrepancy may be explained, in part, by the timing of infection (HRV after allergen) or by the chronicity of allergen exposure. Overall, the dual exposure of sensitized BN to virus and allergen is reminiscent of the severe end of the asthmatic spectrum that is driven by a neutrophilic/Th17 axis (12, 14).

High constitutive *IL33* expression in the Th2^high^ BN is consistent with the heightened susceptibility of this strain to the development of experimental atopic asthma. IL33 is known to promote allergen-driven eosinophil-associated airways inflammation in rodents (50) and *IL33* polymorphisms are associated with wheezing, asthma, and atopy in humans (51). The elevated baseline level of *IL33* in animals expressing the Th2^high^ immunophenotype is a novel finding, given that high *IL33* expression in this context has previously been reported only after relevant immune stimulation (52–54). Based on previous findings (55), it is plausible that high baseline *IL33* expression in BN could be responsible for the attenuation of type I IFN/neutrophilic responses that were observed in this strain following vMC_0_ infection. Surprisingly, allergen exposure of sensitized/virally infected BN appears to have disrupted their *IL33* expression, and it is possible that this may have allowed their type I IFN response to go into overdrive. Although, we were not able to identify the factor involved in inhibiting IL33, a previous study showed that IFNG is a negative regulator of the IL33 pathway (56). In addition, previous studies (32) suggest the lung microenvironment drives innate immune cell function, suggesting that the high expression of IL33 in BN and their altered innate immune response to virus may be driven by an altered lung microenvironment, including the epithelium, which is known to play a key role in the antiviral response (57, 58).

Our findings contrast with the study by Lynch et al. (55) showing that *IL33* expression was upregulated in a mouse model of RSV infection with co-exposure to allergen. This discrepancy may be partly due to differences between species and/or in the kinetics/exposure models themselves. In particular, Lynch et al. focused on the impact of viral infection on the sensitization process during the early postnatal period (55) during which maturation of immune functions is incomplete. In contrast, our study focused on immunologically mature adult animals and was designed to investigate the impact of viral infection on experimental asthma exacerbations in sensitized animals. Notably, allergen challenge was only administered post-viral infection, contrary to other studies (19, 25, 26).

The Th2^high^ BN in our rat model of vMC_0_ infection also differs to previous naive Th2^high^ Balb/c mouse models of HRV infection, which are characterized by acute neutrophilic inflammation (19–22). Our results suggest that BN, unlike Balb/c mice and PVG rats, have an intrinsic defect in their antiviral immune response, similar to a subset of human atopic asthmatics (5–8). Given that asthma in humans is a heterogeneous disease with multiple endotypes/subphenotypes (12–14), it is possible that BN are representative of the extreme end of the Th2^high^ spectrum, with a low neutrophilic/type I IFN responses at baseline and heightened Th2 bias (5, 6), whereas the Balb/c model may represent a more mixed granulocytic Th2 responder phenotype (14). Our rat model, therefore, complements previous mouse models shedding light on different responder phenotypes within the Th2^high^ spectrum.

In conclusion, our experimental animal model suggests that viral infections and sensitization/allergen exposure can potentially interact synergistically and thereby contribute to the hyper-susceptibility to infection-associated airways inflammation characteristic of the Th2^high^ immunophenotype, and that underlying aspects of this phenotype unrelated to “allergic” mechanisms may also contribute independently to their enhanced susceptibility to virus-associated inflammation. Future studies will need to be directed at deciphering the precise underlying mechanisms that drive interactions between antiviral immunity in individuals on the Th2^high^ spectrum and parallel responses to allergens resulting in enhanced airways inflammation, focusing on recruited BAL cells and the airway epithelium. Thus, extrapolation of our findings to human atopic asthmatics suggests potential targeting of aeroallergen-specific immunity in human atopics for prevention of virus-associated severe asthma exacerbations may provide a plausible scientific rationale for therapeutics.

## Ethics Statement

This study was carried out in accordance with the recommendations of the National Health and Medical Research Council of Australia guidelines. The protocol was approved by the Telethon Kids Institute animal ethic committee.

## Author Contributions

AB, PH, and DS designed and supervised the study. J-FL-J, AJ, KM, and JT performed the experiments. J-FL-J, AJ, AB, and DS analyzed the data. LR contributed to project design, methodology, and discussions on data interpretation. J-FL-J, AJ, AB, PH, and DS wrote the manuscript. All authors reviewed the final manuscript.

## Conflict of Interest Statement

The authors declare that the research was conducted in the absence of any commercial or financial relationships that could be construed as a potential conflict of interest.
