# Supplementary material for: Atopy-Dependent and Independent Immune Responses in the Heightened Severity of Atopics to Respiratory Viral Infections: Rat Model Studies
Source: Front Immunol. 2018 Aug 13;9:1805. doi: 10.3389/fimmu.2018.01805 (PMC6099265; doi:10.3389/fimmu.2018.01805)
Supplement: Supplementary file 1 [file Presentation_1.pptx]

## Slide 1
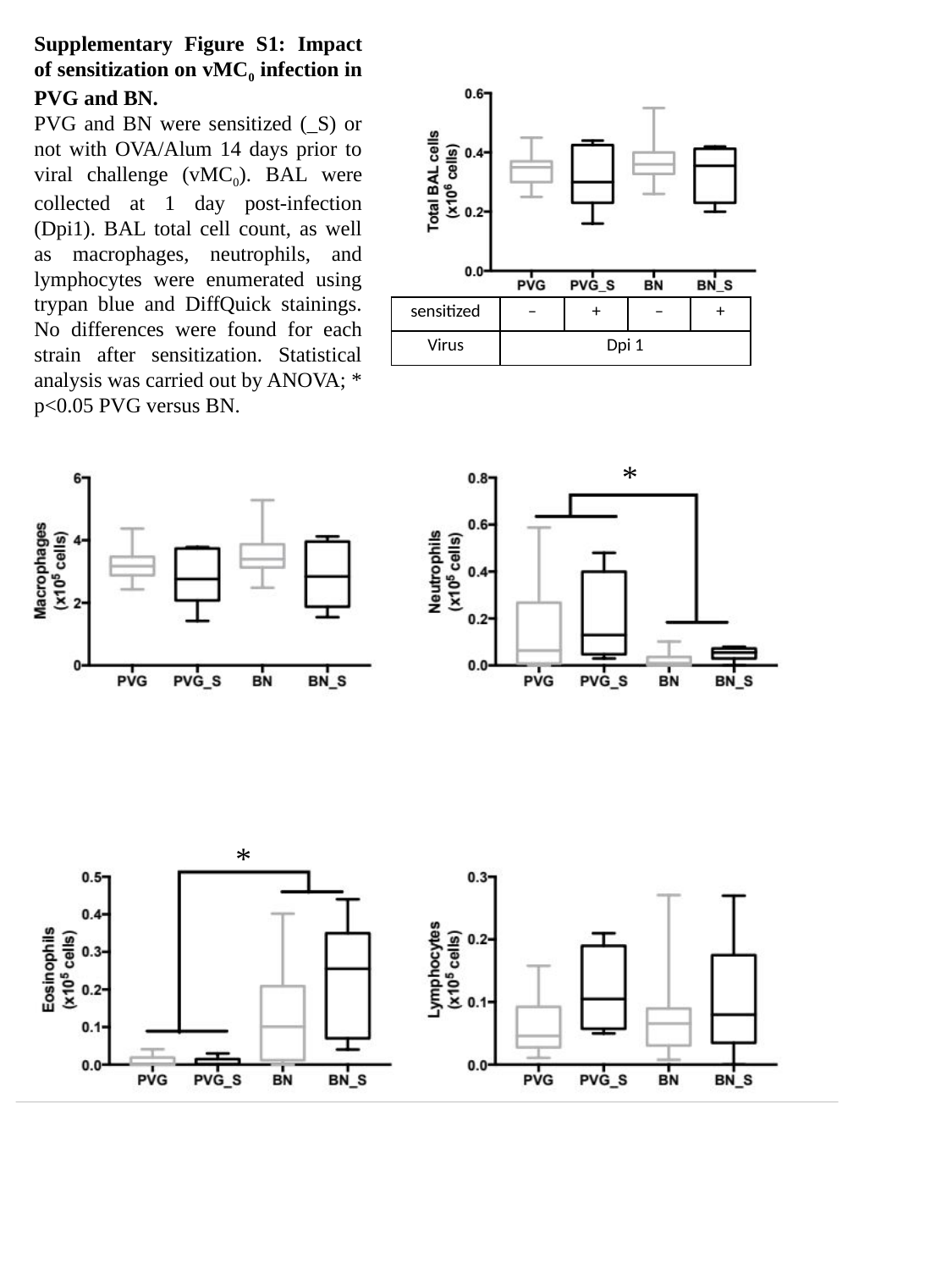

Supplementary Figure S1: Impact of sensitization on vMC0 infection in PVG and BN.
PVG and BN were sensitized (_S) or not with OVA/Alum 14 days prior to viral challenge (vMC0). BAL were collected at 1 day post-infection (Dpi1). BAL total cell count, as well as macrophages, neutrophils, and lymphocytes were enumerated using trypan blue and DiffQuick stainings. No differences were found for each strain after sensitization. Statistical analysis was carried out by ANOVA; * p<0.05 PVG versus BN.
| sensitized | – | + | – | + |
| --- | --- | --- | --- | --- |
| Virus | Dpi 1 | | | |
*
*

## Slide 2
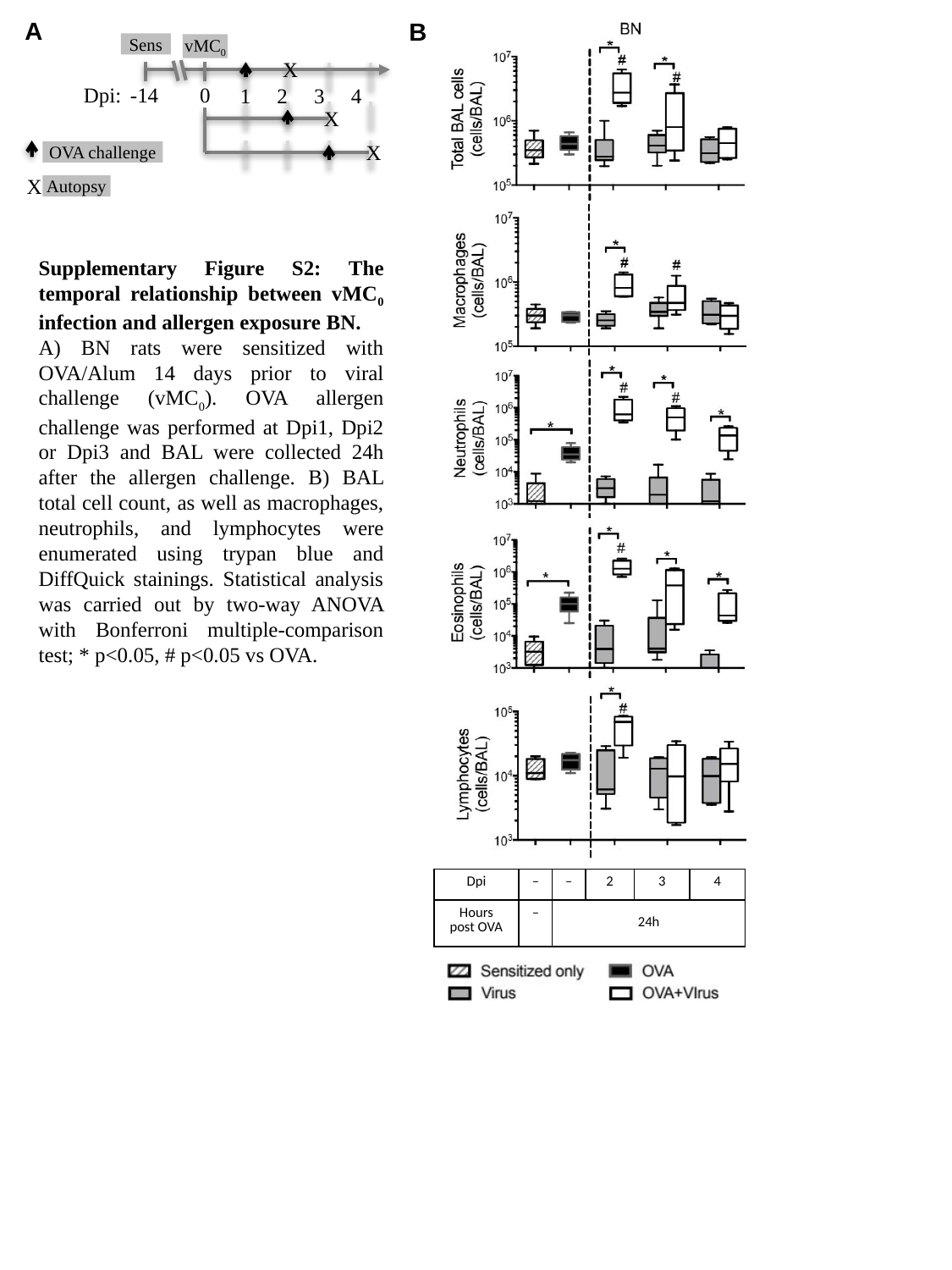

A
B
Sens
vMC0
X
Dpi:
-14
0
1 2 3 4
X
X
OVA challenge
X
Autopsy
Supplementary Figure S2: The temporal relationship between vMC0 infection and allergen exposure BN.
A) BN rats were sensitized with OVA/Alum 14 days prior to viral challenge (vMC0). OVA allergen challenge was performed at Dpi1, Dpi2 or Dpi3 and BAL were collected 24h after the allergen challenge. B) BAL total cell count, as well as macrophages, neutrophils, and lymphocytes were enumerated using trypan blue and DiffQuick stainings. Statistical analysis was carried out by two-way ANOVA with Bonferroni multiple-comparison test; * p<0.05, # p<0.05 vs OVA.
| Dpi | – | – | 2 | 3 | 4 |
| --- | --- | --- | --- | --- | --- |
| Hours post OVA | – | 24h | | | |
